# Supplementary material for: Carbohydrate Metabolism Parameters of Adult Glial Neoplasms According to Immunohistochemical Profile
Source: Biomedicines. 2022 Apr 27;10(5):1007. doi: 10.3390/biomedicines10051007 (PMC9138280; doi:10.3390/biomedicines10051007)
Supplement: Supplementary file 1 [file biomedicines-10-01007-s001.zip › biomedicines-1671866-supplementary.pdf]

Supplementary information

Additional file 1 of « CARBOHYDRATE METABOLISM PARAMETERS OF ADULTS GLIAL NEOPLASMS ACCORDING TO IMMUNOHISTOCHEMICAL PROFILE»

Supplementary Table S1. Clinicopathologic features of gliomas patients

| Characteristics | Grade I (n=1) | Grade II (n=6) | Grade III (n=3) | Grade IV (n=10) |
|---|---|---|---|---|
| Ages (years) | | | | |
| < 60 | 1 | 2 | 2 | 3 |
| ≥ 60 | | 4 | 1 | 7 |
| Gender | | | | |
| Male | | 2 | 2 | 7 |
| Female | 1 | 4 | 1 | 3 |
| Average tumor volume ($cm/^3$) | 121,9 | 99,06 | 66.6 | 89,7 |
